# Dysphagia revealing diffuse idiopathic skeletal hyperostosis: report of two cases and literature review

**DOI:** 10.11604/pamj.2019.32.189.18561

**Published:** 2019-04-17

**Authors:** Monia Ghammam, Jihene Houas, Mouna Bellakhdher, Mohamed Abdelkefi

**Affiliations:** 1 ENT Department and Cervical Surgery Farhat Hached Hospital, Medicine University, Sousse, Tunisia

**Keywords:** Musculoskeletal disorder, ligaments, calcification, dysphagia

## Abstract

Diffuse Idiopathic Skeletal Hyperostosis (DISH) also known as Forestier's disease, is a musculoskeletal disorder characterized by the calcification of ligaments essentially the vertebral longitudinal anterior ligament. Men are generally affected. It is often asymptomatic. The most common extra-spinal clinical manifestation of this disease presents as dysphagia followed by respiratory disturbances such as dyspnea and sleep apnea. In this paper we discuss two cases where the patients have experienced progressive dysphagia. Radiological findings were compatible with DISH. The management was based on diet modification and anti-inflammatory medication.

## Introduction

Diffuse idiopathic skeletal hyperostosis (DISH), also known as Forestier's disease, is a common disorder of unknown etiology, although some correlations with diabetes mellitus, obesity, and age have been noted [1]. It consists in a systemic non inflammatory disease primarily affecting the spine. Involvement of the cervical spine is less frequent than involvement of the lumbar or thoracic one, although it is not rare [2]. It is also associated with the ossification/calcification of tendon, ligament and capsule insertions (entheses) occurring at multiple peripheral sites. Most patients with DISH are asymptomatic. If symptoms are present, they are usually mild in nature and can include back pain, axial stiffness, dyspnea and dysphagia [1, 2]. The aim of our study was to report two cases of DISH revealed by dysphagia.

## Patient and observation

**Figure 1:**
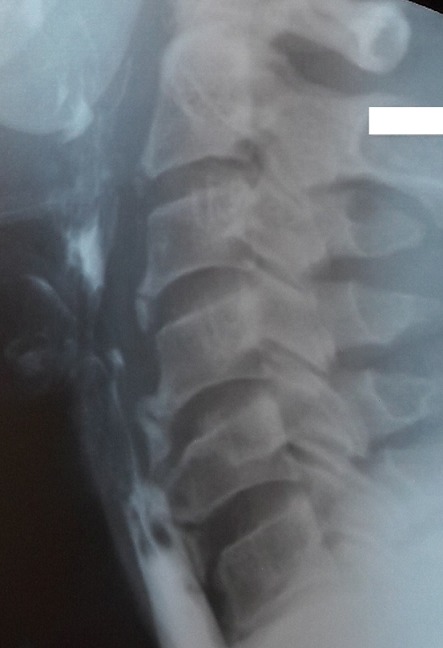
Barium swallow revealing the compression of the oesophagus at the level of C5-6

**Case 1**: a 63-year-old man with a history of type 2 diabetes and arterial hypertension, was referred to our department for a gradually progressive dysphagia involving solid food for seven months, associated with neck pain. There were no other complaints, especially dyspnea, voice changes or weight loss. Physical examination and laboratory findings were unremarkable. The neurological exam was found to be normal. Lateral cervical plain radiographs showed ossification along the anterior aspect of the cervical spine from C2-C7 which was more prominent at C5-C6. A barium swallow study demonstrated compression of the oesophagus at the level of C5 with delayed deglutition Figure 1. We discharged the patient with advice to take semi-solid food in small quantity and at frequent intervals.

**Figure 2:**
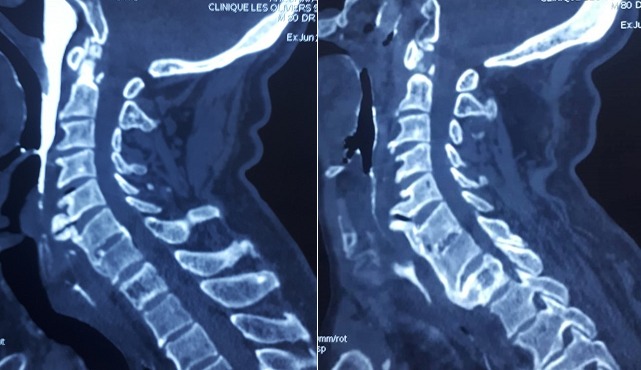
Sagittal cervical CT scans showing diffuse anterior hyperostosis. Arrows pointing to some large osteophytes

**Case 2**: we report a case of an 80-year-old man who presented to our department with 8 months of progressive dysphagia associated with foreign body sensation. There was neither pain during deglutition nor voice change. On examination, no obvious pathology was noticed in oropharynx. There was no palpable mass in his neck. Indirect laryngoscopy revealed a protrusion in the posterior hypo pharyngeal wall, whereas the endolarynx exam was unremarkable with normal vocal cord mobility. The neurological examination was normal. Laboratory results weren't significant. On CT cervical spine, there was extensive flowing anterior osteophytes from the level of C5 to D1. Level of compression on swallow studies was C5-C6 Figure 2. Surgery proposal has been rejected by the patient and thus he has been discharged with anti-inflammatory drugs and was asked to avoid solid food.

## Discussion

DISH, also known as Forestier's disease, was first described by Forestier and Rotès-Querol in 1950. It is a non-inflammatory disorder with an unknown etiology which occurs among the elderly. Men are more often affected [1-3]. The thoracic spine is most commonly involved followed by the lumbar and cervical spines. The pathogenesis of this syndrome is unknown. However, a wide range of factors have been considered in the development of this disease. The condition has potential associations with type II diabetes, obesity, hyperinsulinemia, hyperuricemia and hypercholesterolemia. Patients with DISH are often asymptomatic, however, others can present with back and/or neck pain, restriction of mobility of the spine, peripheral joint affection, dyspnea, hoarseness, stridor, sleep apnea and dysphagia of varying intensities. Neurological deficits are rare in this disease [1, 4-8]. Mechanical compression, periesopharyngeal inflammation with edema and fibrosis and cricopharyngeal spasm are the main mechanisms explaining the genesis of the dysphagia [1, 2]. There are no diagnostic laboratory findings, but evaluation may exclude other differential diagnoses. Plain radiographs are necessary to establish the diagnosis of DISH. The diagnostic criteria are an ossification within the anterior longitudinal ligaments of at least four continuous vertebral bodies with the preservation of disc space height and the absence of degenerative changes [1, 9]. Axial and reconstructed sagittal CT images are essential to determine the true size of the osteophytes and their side of projection. A cervical MRI (Magnetic resonance imaging) is necessary for the detection of intra-canal pathologies [2, 3, 6]. The differential diagnosis includes oropharyngeal tumors, retropharyngeal abscesses, Zenker's diverticulum, esophageal tumors mormotility disorders and other cervical mass lesions. Conservative management options for DISH include diet modification and physical therapy. Anti-inflammatory, anti-reflux, muscle relaxant and sedative medications are also indicated [1, 2]. In case of refractory dysphagia, enteral feeding and gastrostomy may be indicated. Surgical treatment should be selected with care. It is indicated in case of failure of conservative management, increased dysphagia with important weight loss and upper airway obstruction. It is performed via the anterior (Smith-Robinson approach) and trans pharyngeal approaches [5, 10-12]. The resection of the osteophytes is performed by creating a long incision with adequate exposure. Tracheostomy may be indicated in acute respiratory distress [8, 9]. Severe complications of the surgical treatment such as hematoma, recurrent nerve palsy, esophageal injury and Horner syndrome are to be considered [5].

## Conclusion

DISH is an uncommon cause of dysphagia mostly affecting old individuals with male preponderance. It produces non marginal osteophyte formation in the spine that often results in ankylosis. Although often asymptomatic, patients may develop dysphagia in rare cases. While conventional radiography clearly confirms the diagnosis, CT and MRI better detect associated findings. Conservative management is recommended first. However, when conservative management fails to alleviate severe symptoms, surgical management is indicated.

## Competing interests

The authors declare no competing interests.
